# The utilization of guidewires for adjusting the intraoperative catheter malposition during the venous access port implantation: A retrospective study

**DOI:** 10.1097/MD.0000000000040461

**Published:** 2024-11-08

**Authors:** Li Zhang, Jingjin Wu

**Affiliations:** a Department of Nephrology, The Fourth Affiliated Hospital of School of Medicine, and International School of Medicine, International Institutes of Medicine, Zhejiang University, Yiwu, China; b Department of General Surgery, The Fourth Affiliated Hospital of School of Medicine, and International School of Medicine, International Institutes of Medicine, Zhejiang University, Yiwu, China.

**Keywords:** guidewire, malposition, operation, technique, TIVAP

## Abstract

This study presents an in-depth exploration of various adjustment methods for intraoperative catheter malposition by guidewires in the implantation of totally implantable venous access ports (TIVAP). It not only aims to summarize these methods but also endeavors to identify the most advantageous approach. The patient list was searched using the hospital information system from January 1, 2022, to October 31, 2023. All patients who had undergone chest port placement using the axillary vein (AxV) or subclavian vein (SCV) approach were reviewed, and further imaging was performed to confirm the guidewire applied to adjust the guidewire or catheter from the internal jugular vein into the superior vena cava (SVC) under fluoroscopy. Demographic data, diagnoses, technical outcomes, and perioperative complications were collected. About 32 patients with an average age of 62.8 years were included in the study. The operation time was 29.3 ± 13.3 minutes and SVC selecting time was 16.9 ± 11.5 seconds. The dose of X-ray exposure was only 7.2 ± 9.0 μGym^2^. Adjustments combined the guidewire with the puncture needle had the shortest SVC selection time and operation time with a minimal radiation dose. intraoperative catheter malposition can be timely and effectively adjusted using guidewires under fluoroscopy during any process of TIVAP implantation. Timely adjustment using a guidewire after inserting into the puncture needle is an optimal choice for a smooth and successful operation.

## 1. Introduction

The totally implantable venous access port has been widely used since first reported by Niederhuber et al in 1982,^[1]^ especially for patients with malignant tumors.^[2,3]^ Nowadays most medical centers can carry out the port implantation maturely. While the chest wall ports via the internal jugular vein (IJV) approach are the most widely used,^[4,5]^ the port implantation via the AxV/SCV approach has been on the rise in recent years.^[6]^ Due to the ultrasound-guided technique, the chest wall port via the AxV is becoming more and more mature,^[7–9]^ which greatly reduces the risk of pinch-off syndrome caused by the SCV approach and significantly improves the patient’s comfort and appearance because it avoids the subcutaneous tunnel crossing the collarbone.^[10]^ However, there is still a risk that the guidewire or catheter may easily enter the IJV using the AxV/SCV approach for totally implantable venous access ports (TIVAP) implantation, especially when the angle between the SCV and the ipsilateral IJV is >90°.^[11]^ Typically, most physicians will ask the patient to tilt the head to the operative side or press on the supraclavicular fossa to allow the guidewire or catheter to enter the superior vena cava (SVC).^[12]^ However, in many cases, this method may not obtain good efficiency due to the deep location of the vein. In the field of endovascular therapy, the guidewire can easily help the catheter to arrive into the targeted vessel.^[13–15]^ If the technique of endovascular guidewire application can be utilized in TIVAP implantation, the above problem will be well solved. Under fluoroscopic guidance, the guidewire can enter into the SVC explicitly, which means the success of the TIVAP implantation. In the present study, we will share some experiences of how to dexterously use guidewire in TIVAP implantation in different situations.

## 2. Methods

The patient list was searched from the hospital information system from January 1, 2022, to October 31, 2023. All patients who had undergone chest port placement using the AxV/SCV approach were reviewed and further imaging reviewing was performed to confirm that the guidewire was applied to adjust the guidewire or catheter from the IJV into the SVC under fluoroscopy. The patients using the guidewire to adjust IJV malposition during operations were included. The demographic data, diagnosis, technical outcomes, and perioperative complications were collected. All patients used TIVAP from Bard Access Systems, Inc. The guidewires consisted of the self-contained guidewire in the port system and the Radifocus Guide Wire M (RF*GA35153M, Terumo Vietnam Co., Ltd). The single incision was performed for both the port pocket and vein puncture. The technical outcomes included the method of guide-wire application, operation time, SVC selection time, and X-ray exposure dose. While the early complications consisted of inadvertent arterial puncture, pneumothorax, and hemothorax observed before the first use, the late complications included port dysfunction, device-related infection, catheter-related thrombosis, and catheter malposition. postoperative complications were observed and recorded from the implantation day to the removal day or December 31, 2023. This study was approved by the Ethics Committee of the Fourth Affiliated Hospital, Zhejiang University School of Medicine (No.: K2024004) and was performed following the Declaration of Helsinki. Informed consent was waived due to the retrospective nature of the study.

## 3. Technique for malposition adjustment

**Figure 1.**
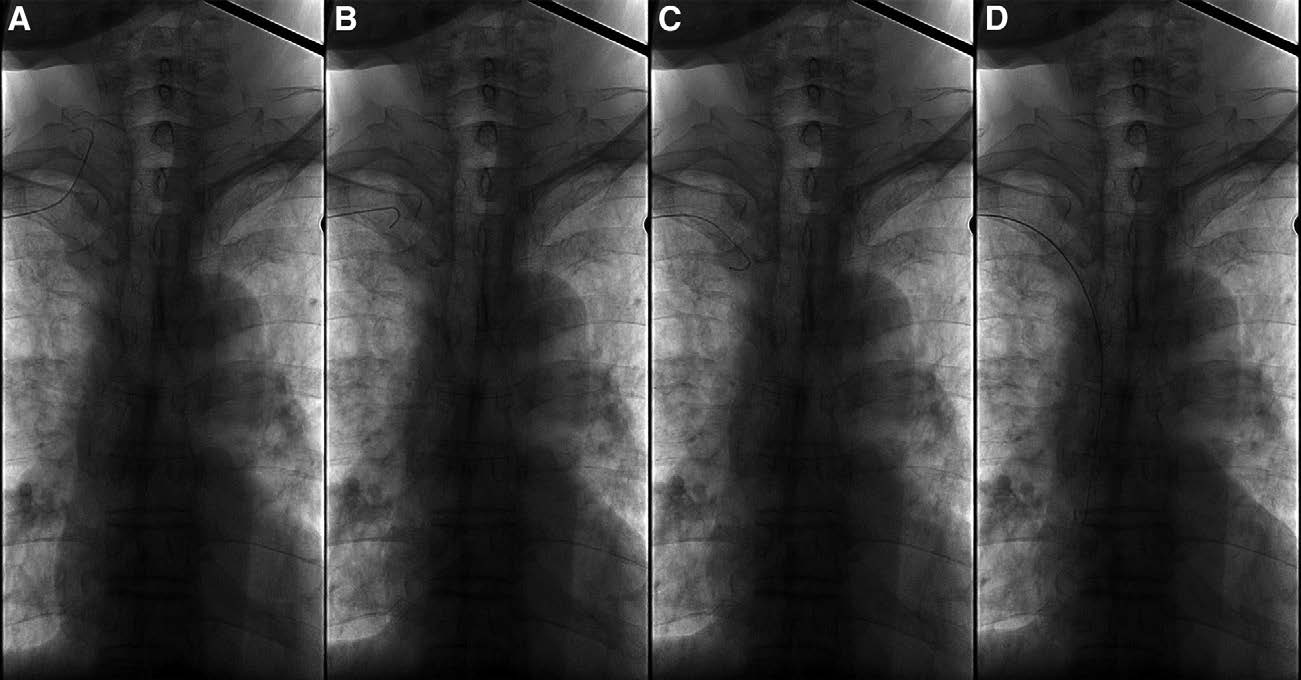
Adjustment combined the guidewire with the puncture needle. (A) The guidewire was confirmed in the internal jugular vein (IJV). (B) Withdraw the guidewire to the SCV. (C) The guidewire was inserted into the brachiocephalic vein under fluoroscopy. (D) The guidewire was delivered into the cavo-atrial junction.

When the puncture needle is not removed, the tip of the guidewire can be adjusted under fluoroscopy to select into the SVC (Fig. 1).

**Figure 2.**
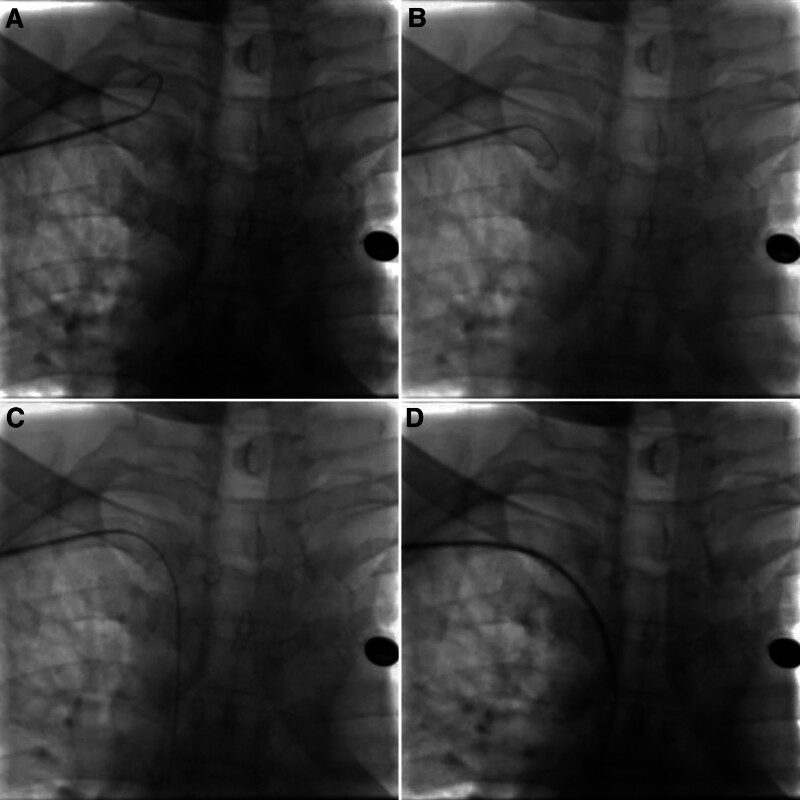
Adjustment combined the guidewire with the sheath. (A) The guidewire was confirmed in the IJV. (B) Withdraw the guidewire and the sheath to the SCV. (C) The guidewire was inserted into the superior vena cava (SVC) under fluoroscopy. (D) The sheath was delivered into the SVC followed the guidewire.

When the catheter sheath has been placed, the catheter sheath should be withdrawn to the SCV, and then the guidewire can be selected for the SVC under fluoroscopy (Fig. 2). If unsuccessfully, the guidewire tip can be properly molded to be convenient for the guidewire entering the SVC.

**Figure 3.**
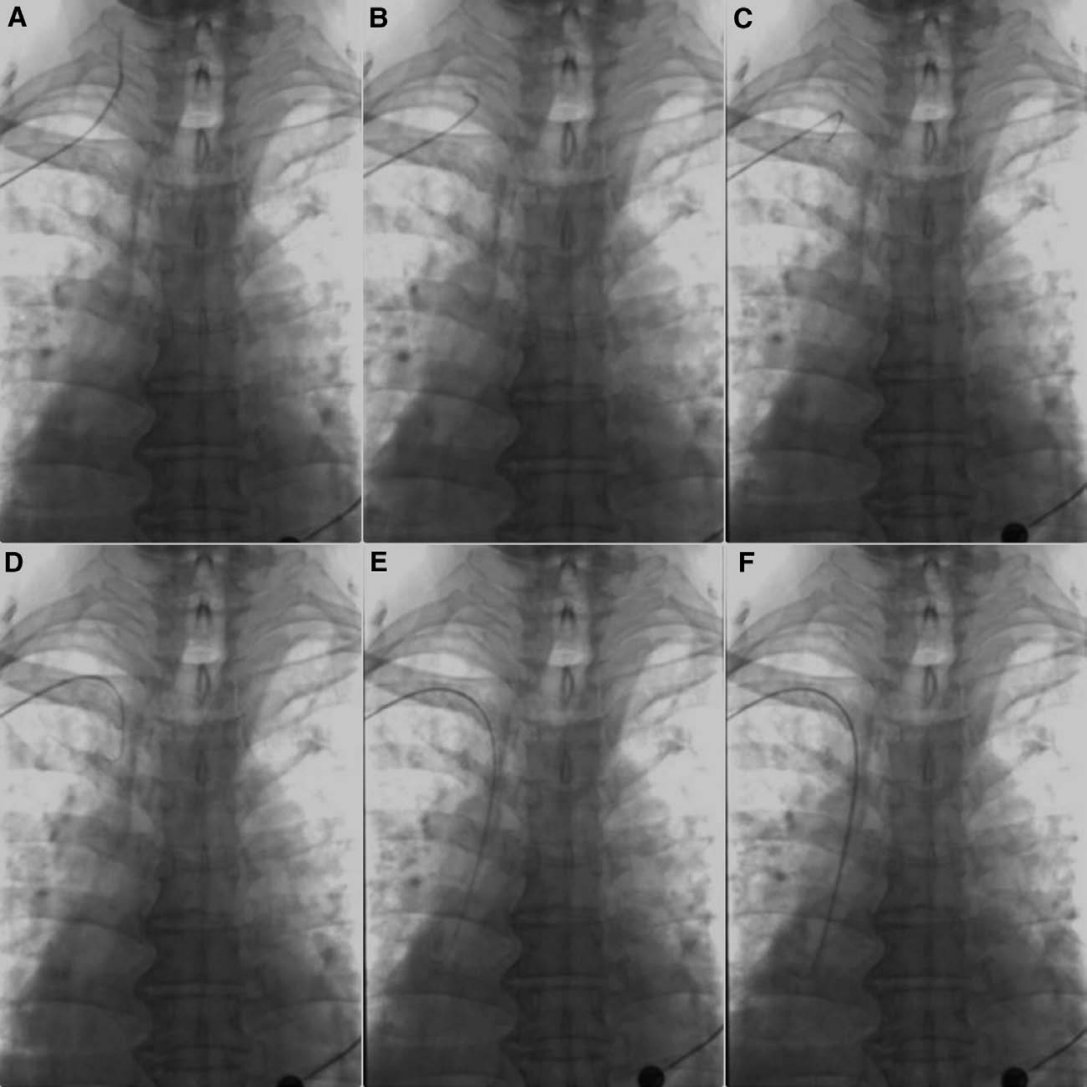
Adjustment combined the guidewire with the catheter. (A) The catheter was confirmed in the IJV. (B) Withdraw the catheter to the SCV. (C) Withdraw the guidewire to the SCV. (D) The guidewire was inserted into the brachiocephalic vein under fluoroscopy. (E) The guidewire was delivered into the cavo-atrial junction. (F) The catheter was delivered into the SVC followed the guidewire.

When the catheter has been placed, the guidewire can pass inside the catheter and be selected into the SVC. At last, the catheter can be sent into the SVC following the guidewire smoothly (Fig. 3).

Descriptive statistics were used to analyze this case cohort and mean ± standard deviation was used for count variables. The data were analyzed by SPSS v27.

## 4. Results

There were 539 TIVAP implantations searched and 32 of them with an average age of 62.8 years, were included in the study. All surgeries were successfully performed. Male patients accounted for the majority (21/32) of patients. Only 2 cases underwent the SCV approach, and most ports were implanted in the right anterior chest wall (Table 1). The mean operation time was 29.3 ± 13.3 minutes and the SVC selection time was 16.9 ± 11.5 seconds. The mean dose of X-ray exposure was only 7.2 ± 9.0 μGym^2^ (Table 2). The operation time, SVC selection time, and X-ray exposure of each method were summarized in Table 3. The first case of adjustment with radifocus guidewire had primarily used traditional methods but with little effect. Subsequently, the radifocus guidewire passed through the catheter and was selected into the SVC. Inadvertent arterial puncture occurred in only 1 case and pneumothorax/haemothorax didn’t happen. None of the port dysfunction, device-related infection, or catheter-related thrombosis was observed. Catheter malposition occurred in 2 cases after the first use of TIVAP and the 2 patients rejected any adjustment. Fortunately, their ports maintained normal function without catheter-related thrombosis.

**Table 1:** Main clinic information of patients.

Age (yr)	62.8 ± 11.5
Gender, *n*	
Male	21
Female	11
Height (cm)	162.5 ± 7.2
Weight (kg)	62.8 ± 8.6
Pathology, *n*	
Gastric cancer	8
Lung cancer	7
Breast cancer	5
Colorectal cancer	7
Nasopharyngeal cancer	1
Cholangiocarcinoma	1
Liver cancer	1
Pancreatic cancer	1
Ovarian cancer	1

**Table 2:** Perioperative information and complications.

Punctured vessels, n	
Right AxV	26
Right SCV	2
Left AxV	4
Method of guide-wire application, n	
Catheter-radifocus guidewire	7
Catheter-self-contained guidewire	1
Sheath-self-contained guidewire	3
Puncture needle-self-contained guidewire	21
Operation time (min)	29.3 ± 13.3
Time for SVC selection (s)	17.3 ± 11.5
Exposure dose (μGym^2^)	7.2 ± 9.0
Catheter days (d)	169.9 ± 95.0
Early complications, n	
Inadvertent arterial puncture	1
Late complications, n	
Catheter malposition	2

**Table 3:** Intraoperative information of different methods.

	Catheter-radifocus guidewire, n* = *7	Catheter-self-contained guidewire, n* = *1	Sheath-self-contained guidewire, n* = *3	Puncture needle-self-contained guidewire, n* = *21
Operation time (min)	40.7 ± 16.9	25.0	27.0 ± 3.0	26.0 ± 11.3
Time for SVC selection (s)	26.5 ± 12.6	31.1	25.1 ± 6.3	12.4 ± 8.9
Exposure dose (μGym^2^)	11.3 ± 10.5	9.0	6.4 ± 5.2	5.8 ± 9.0

## 5. Discussion

There are many vascular accesses for TIVAP implantation, the most commonly reported being the IJV.^[5,16,17]^ In recent years, an increasing number of port implantations through the AxV/SCV have been reported.^[9,18]^ In addition, the single-incision technique has been widely applied in chest wall infusion port implantation by many surgeons. As its name implies, a single incision can avoid discomfort caused by subcutaneous tunnels crossing in front of the clavicle while achieving minimal wound and aesthetical appearance.^[8,19]^ Some literature has reported no statistical difference in perioperative complications between IJV and AxV/SCV access.^[6,20]^ Therefore, our center currently adopts the single-incision technique in most TIVAP implantation.

In this study, the majority of patients underwent access via the right AxV. There are several possible rationales for this choice. Firstly, certain studies have demonstrated that there is no statistically significant difference in complications between the left AxV approach and the right AxV approach.^[9,19]^ Secondly, puncturing the left AxV/SCV may pose a risk of damaging the thoracic duct.^[21]^ Thirdly, if a successful puncture of the right AxV/SCV cannot be achieved during the operation, the right IJV approach can serve as an alternative. This approach is closer to the SVC compared to the left IJV approach. Fourthly, the majority of our surgeons are right-handed, and performing the operation on the right side of the patient is generally more seamless.

To our knowledge, the primary catheter malposition rate may range from 2.9% to 13.1%, which was mostly confirmed by postoperative X-ray chest radiography.^[22–24]^ However, catheter malposition may also occur during the period between surgery and chest X-ray examination. In effect, the accurate incidence of intraoperative IJV malposition under the AxV/SCV approach was lack of data. The incidence of catheter malposition during peripherally inserted central catheter (PICC) placement has been reported to range from 6.63% to 24.6%,^[25,26]^ with the rate of malposition in the IJV ranging from 3.31% to 12.3%, and 72.73% of catheter malposition occurrences were in the IJV, as reported by Song et al.^[12]^ Catheters are required to enter the SVC from the SCV during both PICC placement and TIVAP implantation via the AxV/SCV approach. In the present study, the guidewire or catheter was observed inside the IJV in 5.94% (32/539) patients, which is in line with the other research.

The catheter or guidewire unexpectedly located in the IJV may occur at any time during TIVAP implantation under AxV/SCV access. In the earlier cases, once we found that the catheter was ectopically located in the IJV, we tried various adjustment methods, including advising the patient to tilt the head to the port side, keeping the jaw close to the shoulder, or compressing the IJV above the clavicle, but they did not achieve perfect results. A radifocus guidewire was then used to pass through the catheter and enter into the SVC, followed by the catheter smoothly delivered to near the cavo-atrial junction. Later, we routinely performed fluoroscopy after inserting the guidewire, and once the guidewire was found located in the IJV, the guidewire was timely applied to be selected into the SVC under fluoroscopy to minimize the small but serious risk of vessel perforation because of repeatedly blind withdrawal and push of the guidewire tip towards to the vein wall.^[27]^ In the present study, using a self-contained guidewire through the puncture needle to select the SVC was the fastest. During the process, the patients didn’t need to do their part and the operation time was shortened because of the quicker and successful procedure.

According to the Chinese basic standards for protection against ionizing radiation and for the safety of radiation sources, the dose limit for occupational exposure shouldn’t exceed 20 mSv per year.^[28,29]^ The natural radiation is about 2.4 mSv per year.^[30,31]^ In the present study, The dose of X-ray exposure was only 7.2 ± 9.0 μGym^2^, which was far less than the dose limit.^[32]^ Therefore, using the guidewire to choose the SVC under fluoroscopy is safe for both surgeons and patients.

Because there was no accessible intracardiac electrocardiogram monitoring, intraoperative fluoroscopy was performed to confirm the catheter tip position at our center. Based on our understanding, the flexible use of the guidewire in TIVAP implantation has many advantages. First, the guidewire tip with an angle that is visible and accurately localized under fluoroscopy has a high success rate of selecting target vessels in the endovenous cavity. It can not only minimize the damage to the venous lining by repeatedly pushing the guidewire under a non-fluoroscopic state but also avoid the uncertainty of blind adjustment. Second, by adjusting the X-ray exposure area, the patient and the operator can suffer from little X-ray exposure during the operation.

To the best of our knowledge, this study comprehensively introduced different strategies of guidewire application for intraoperative catheter malposition in TIVAP implantation. The above-mentioned has amply demonstrated that flexibly manipulating guidewires can make operation smooth, easy, and safe. Nevertheless, we must recognize the following shortcomings of this study. Firstly, this study is a retrospective single-center study, so there may be bias in the selection of patients, and the superiority or inferiority of our shared methods and other methods of IJV malposition adjustment still needs to be proved by a large-sample, multicenter randomized controlled clinical trial. Secondly, the number of cases in this study was small, and large-sample, multicenter randomized controlled clinical trials are needed. Third, the above guidewire utilizing strategies do not represent all the guidewire application scenarios, and more methods of intraoperative adjusting catheter malposition are awaited.

## 6. Conclusion

Catheter malposition cannot be eliminated during TIVAP implantation. The application of various types of guidewires will facilitate intraoperative adjustment for catheter malposition, especially for port implantation via AxV/SCV access. Timely adjustment using a guidewire after inserting into the puncture needle is an optimal choice for a smooth and successful operation.

## Author contributions

**Conceptualization:** Jingjin Wu.

**Data curation:** Li Zhang, Jingjin Wu.

**Formal analysis:** Jingjin Wu.

**Funding acquisition:** Li Zhang, Jingjin Wu.

**Methodology:** Jingjin Wu.

**Supervision:** Jingjin Wu.

**Writing – original draft:** Li Zhang, Jingjin Wu.

**Writing – review & editing:** Jingjin Wu.
